# Potential Involvement of *Buchnera aphidicola* (Enterobacteriales, Enterobacteriaceae) in Biotype Differentiation of *Sitobion avenae* (Hemiptera: Aphididae)

**DOI:** 10.3390/insects15120980

**Published:** 2024-12-11

**Authors:** Yanyan Lan, Jingpeng Li, Shuo Zhang, Qiuju Qin, Deguang Liu, Chen Luo, Shipeng Han, Da Wang, Yunzhuan He

**Affiliations:** 1 College of Plant Protection, Hebei Agricultural University, Baoding 071001, China; yanyanlan1@163.com (Y.L.); 13754518565@163.com (J.L.); 15100518136@163.com (S.Z.); qiujuqin@163.com (Q.Q.); 2 College of Plant Protection, Northwest A&F University, Yangling 712100, China; dgliu@nwafu.edu.cn (D.L.); chen.luo@nwafu.edu.cn (C.L.); 3 College of Plant Protection, Shandong Agricultural University, Tai’an 271000, China; hanshipeng1994@163.com

**Keywords:** artificial diets, biotype differentiation, *Buchnera* abundance, genetic variation, grain aphid, RNA-seq

## Abstract

*Buchnera aphidicola*, an obligate endosymbiont of most aphid species, can influence aphids’ host adaptability through amino acid metabolism, potentially mediating biotype differentiation. However, its role in the biotype differentiation of *Sitobion avenae* remains unclear. To address this issue, six *S. avenae* biotypes were tested in this study. *Buchnera* abundance varied among biotypes fed on different wheat/barley varieties (i.e., Zhong 4 wumang, 186-TM12-34; Dulihuang, Zaoshu No.3, Xiyin No.2). The reduction in *Buchnera* abundance through antibiotic (rifampicin) treatment altered the virulence of five *S. avenae* biotypes. Based on transcriptome analysis, the differential expression of three genes (i.e., *LeuB*, *TrpE*, and *IlvD*) related to leucine, tryptophan, isoleucine, and valine metabolism was detected between different biotypes. Principal component analysis showed that leucine and tryptophan deficiencies most significantly impacted nymph development duration and aphid fecundity. Additionally, a neighbor-joining phylogenetic tree indicated the genetic differentiation of *Buchnera* among different biotypes. These results suggest *Buchnera*-mediated amino acid metabolism is correlated with biotype differentiation in *S. avenae*, although the precise mechanisms by which *Buchnera* influences this differentiation require further investigation. This study can offer a theoretical basis for the development of resistant crops, leading to the sustainable control of this aphid and reduced reliance on chemical insecticides.

## Simple Summary

This study explored the role of *Buchnera aphidicola*, an obligate endosymbiont, in the biotype differentiation of *Sitobion avenae* (Fabricius). *Buchnera* abundance varied among biotypes when fed on different wheat and barley varieties. The reduction of *Buchnera* abundance by antibiotic treatment altered the virulence of the five biotypes. Transcriptome analysis showed that genes involved in amino acid metabolism were differentially expressed between different biotypes of *S. avenae*. The deficiencies in leucine and tryptophan most significantly affected nymph development and aphid fecundity. The genetic differentiation of *Buchnera* among *S. avenae* biotypes was also observed. These findings suggest that *Buchnera* may play a role in the biotype differentiation of *S. avenae* through its influence on amino acid metabolism. This provides valuable insights for breeding resistant crop varieties and reducing the reliance on insecticides.

## 1. Introduction

Utilizing the resistance of host plants to control insect pests serves as an effective control strategy, providing cost savings, supporting biocontrol efforts, and decreasing the reliance on potentially harmful insecticides [1]. The rapid emergence of new insect biotypes poses significant challenges to the effectiveness of resistant crops in pest management. For example, the resistance of wheat varieties with the *Dn4* gene deployed in the United States in 1995 was overcome by a new biotype (RWA2) of *Diuraphis noxia* (Mordvilko) just eight years later [2]. Insect biotypes are generally described as groups within a species that can overcome the resistance of cultivated plants, which are effective against other groups of the same species. Around half of all insects exhibiting biotype differentiation are members of the Aphididae family [3]. Biotype divergence has been identified in numerous aphids that cause significant harm to agricultural production, including *Aphis glycines* (Matsumura), *D. noxia*, and *Acyrthosiphon pisum* (Harris) [4,5,6,7]. The finding that aphids are more prone to biotype divergence than other insects may be closely related to their parthenogenetic reproduction and, potentially, to the influence of endosymbionts [4,8,9].

*Buchnera aphidicola* (Enterobacteriales, Enterobacteriaceae), as a vital obligate endosymbiont, is distributed in most aphids [10,11]. While *Buchnera* provides essential amino acids (EAAs) and other nutrients critical for aphid survival and reproduction [12,13], it may also influence aphid feeding preferences and plant specialization through the modulation of amino acid metabolism, potentially contributing to biotype differentiation in aphids [1]. Recent studies provide evidence suggesting this possibility. For example, *Buchnera* can provide *A. glycines* biotype 2 with a more sufficient supply of EAAs, resulting in the ability of this biotype to overcome the resistance of soybean variety with the gene *Rag1* [14,15]. Subsequent genome sequencing of *Buchnera* in *A. glycines* revealed that 19 SNPs were identified between biotypes 1 and 2, many of which were located in genes related to the metabolism of amino acids [16]. Meanwhile, many studies have indicated that the divergence in the amino acid metabolism of *Buchnera* in different biotypes of the same aphid might be related to the abundance and genetic variation of *Buchnera* [17,18,19].

In recent years, some studies have attempted to ascertain the specific amino acid metabolism and key genes involved in *Buchnera*-mediated aphid biotype divergence. Leucine and arginine metabolism were considered to be the critical amino acid metabolic pathways in the biotype differentiation of *D. noxia* and *A. pisum*, respectively [17,20]. This distinction may be related to the different compositions of free amino acids in the host plants that different species of aphids feed on, and another reasonable explanation is that significant genetic variations of *Buchnera* existed in different species of aphids [21,22]. So far, the molecular mechanism for the biotype differentiation of aphids mediated by *Buchnera* is still not fully elucidated, and the specific genes involved often remain unidentified.

The grain aphid *Sitobion avenae* (Fabricius) (Hemiptera: Aphididae) is a significant worldwide pest inflicting substantial economic losses in agriculture [23,24,25]. In our earlier research, several biotypes of *S. avenae* were identified by examining their specific virulence response profiles on multiple barley/wheat varieties [26]. Insufficient free amino acids in aphid-resistant wheat cultivars are an important factor in conferring resistance [27]. Therefore, we speculate that *Buchnera*, which can provide EAAs for host aphids, may be involved in the biotype differentiation of *S. avenae*. In this research, we characterized the *Buchnera* abundance of six *S. avenae* biotypes fed on different wheat/barley varieties, as well as the changes in the virulence response profiles of *S. avenae* biotypes after antibiotic treatment reduced the abundance of *Buchnera*. The differentially expressed genes of *Buchnera* between different biotypes were detected by RNA-seq. Then, six *S. avenae* biotypes were fed with artificial diets composed of different amino acids, and the differences in the life-history traits among different biotypes were compared. Finally, molecular marker technology was used to analyze the genetic variation of *Buchnera* between different *S. avenae* biotypes. The specific objectives are to (1) investigate the potential role of *Buchnera* in the biotype differentiation of *S. avenae*; (2) examine the influence of the abundance and genetic variation of *Buchnera* on the biotype differentiation of this aphid; and (3) identify the amino acid metabolism potentially involved in the biotype differentiation of *S. avenae* mediated by *Buchnera*.

## 2. Materials and Methods

### 2.1. Aphid Biotypes

In our previous study, multiple *S. avenae* biotypes (i.e., biotypes 1–5 and 7; Table S1) were identified based on their unique performance profiles on resistant wheat varieties (i.e., Zhong 4 wumang and 186-TM12-34) and barley varieties (i.e., Dulihuang, Zaoshu No.3, and Xiyin No.2). The collection and microsatellite information for all clones of the six biotypes selected in this study are shown in Table S2. Details on primer information and fragment size detection methods for each microsatellite locus are available in our previous study [26]. Under the conditions of 22 ± 1 °C temperature, 65 ± 5% relative humidity, and a 16:8 (L:D) h photoperiod, each aphid clone was reared in isolation on the wheat variety Aikang 58, known to be susceptible to *S. avenae* [26].

### 2.2. Detection of the Abundance of Buchnera in Six S. avenae Biotypes on Different Plants

Before this test, for each biotype, three first-instar nymphs from each clone were mixed together and raised for three generations on Aikang 58, forming a clonal complex of each biotype. One randomly chosen new-born first-instar nymph from the complex of each biotype was transferred onto single seedlings at the two-leaf stage of five resistant wheat/barley varieties (i.e., Zhong 4 wumang, 186-TM12-34, Dulihuang, Zaoshu No.3, and Xiyin No.2) and one susceptible control wheat variety (i.e., Aikang 58). These varieties were used in our previous study to identify *S. avenae* biotypes [26]. Under the above environmental conditions, five 5-day-old nymphs or wingless adults within 24 h were collected for DNA extraction as a single biological replicate. The abundance of *Buchnera* was measured by qRT-PCR, using the ratio of the copy number of the *Buchnera*-specific gene *groEL* to that of the aphid gene *ef1α* [28]. The amplification efficiencies of *groEL* and *ef1α* primers were 1.02 and 0.94, respectively, and their sequences are provided in Table S3. Each qRT-PCR reaction was carried out in a total volume of 20 μL, consisting of 10 μL SYBR Green Master Mix (UElandy, Suzhou, China), 1 μL DNA (100 ng/uL), 0.5 μL each forward and reverse primer (10 μM), and 8 μL ddH_2_O. The qRT-PCR cycling conditions included an initial denaturation at 95 °C for 2 min, then 45 cycles of amplification with denaturation at 95 °C for 10 s, annealing at 54 °C for 30 s, and extension at 72 °C for 30 s. The melt curve analysis at the annealing temperature showed no primer dimers or nonspecific amplification. All qRT-PCR assays were conducted using the CFX96 Real-Time System (Bio-Rad, Hercules, CA, USA). For each biotype under each treatment, four biological replicates and three technical replicates were included. Copy numbers were calculated for each gene in different samples based on a standard curve using the method described by Serteyn et al. [17].

### 2.3. Determination of the Virulence Response Profiles of Six S. avenae Biotypes on Wheat/Barley Varieties Before and After Rifampin Treatments

To minimize the potential influence of facultative endosymbionts in aphids, we selected a clone (i.e., clone 3, Table S2) from each biotype that was devoid of the most commonly characterized facultative endosymbionts for the subsequent tests. The most commonly characterized facultative endosymbionts (i.e., *Hamiltonella defensa*, *Regiella insecticola*, *Rickettsia* sp., *Spiroplasma* sp., *Serratia symbiotica*, *Wolbachia pipientis*) in *S. avenae* and the detection methods were referenced from Hu et al. [29] and Wang et al. [30]. All primer sequences are listed in Table S4. The electrophoresis detection results of facultative endosymbionts in clone 3 of each biotype before 72 h of antibiotic treatment are shown in Figure S1.

Referring to the method of Zhang et al. [31] and Colella et al. [32], rifampicin (Macklin Biochemical Co., Ltd., Shanghai, China) was added to the artificial diets, and the artificial diets were fed to aphids using an aphid feeder. The aphid feeder was constructed by stretching a thin membrane of Parafilm (Bemis, Neenah, WI, USA) over a transparent plastic tube of 30 mm diameter and 30 mm height, with 0.4 mL of the artificial diet (Table S5) added between two layers of Parafilm.

In order to determine the optimal concentration of rifampin that can effectively reduce *Buchnera* abundance and make aphids complete a normal generation, artificial diets containing different concentrations of rifampicin (0 μg/mL, 1 μg/mL, 2 μg/mL, 4 μg/mL, 8 μg/mL, 16 μg/mL) were prepared, and new-born first-instar nymphs (within 24 h) of three biotypes (i.e., biotypes 1–3) were maintained on these artificial diets. After 24 h, fifty nymphs were collected for DNA extraction as a single biological replicate. The abundance of *Buchnera* was detected according to the method described above. At the same time, 24 h after treatment, one aphid was transferred to a single plant seedling of Aikang 58. Test individuals were monitored daily for molting or reproductive events until 10 days after the onset of reproduction for each individual. Each treatment was repeated three times for each biotype.

Based on the above experimental results, the optimum concentration of rifampicin used in the test was determined. New-born first-instar nymphs of the six biotypes were fed with artificial diets containing selected concentrations of rifampicin and artificial diets without rifampicin, respectively. After 24 h, the abundance of *Buchnera* of each biotype was detected according to the above method to verify the effect of rifampicin treatment. Simultaneously, 24 h after antibiotic treatment with or without antibiotics, one aphid was transferred to a single plant seedling of each of the six varieties (i.e., Zhong 4 wumang, 186-TM12-34, Dulihuang, Zaoshu No.3, Xiyin No.2 and Aikang 58). The 10 d fecundities of all aphids were determined according to the method described above. In addition, the count of aphid individuals per plant was recorded 12 days following infestation. Each treatment was repeated four to six times for each biotype. Plant resistance to *S. avenae* was assessed using the aphid counts ratio, calculated as the number of aphids on each variety divided by the number on the susceptible control variety Aikang 58. Following Wang et al. [26], a variety was classified as resistant (R) if the value of the aphid counts ratio was less than 0.9 and as susceptible (S) if the value was 0.9 or greater.

### 2.4. Transcriptome Sequencing and Annotation of the Assembly

Because the virulence profiles of biotypes 1 and 3 differed most among all biotypes, three clones of biotypes 1 and 3 (clone 1–3, Table S2) were selected, and differentially expressed genes of *Buchnera* in these two biotypes were detected based on transcriptome analysis. Neonatal first-instar nymphs were transferred to seedlings at the two-leaf stage of the wheat cultivar Aikang 58 and reared according to the above conditions. Each time, ten wingless adult aphids emerging within 24 h were collected, placed in 1.5 mL RNase-free tubes, quickly frozen in liquid nitrogen, and stored at −80 °C. Each *S. avenae* biotype was subjected to three biological replicates.

Total RNA extraction, cDNA library construction, and quality assessment were performed following the protocol described by Huang et al. [33]. Based on the paired-end DNA sequencing technique, all cDNA libraries were sequenced on the Illumina HiSeq 2500 system (Illumina, Inc., San Diego, CA, USA) provided by Ovidson Gene Technology Co., Ltd., Beijing, China. The raw data had been deposited in the NCBI Sequence Read Archive (SRA) database under Accession ID: PRJNA575173. The software Fastp (v0.21.0) was used to remove low-quality reads (those with more than 50% of nucleotides having a Qphred score ≤ 20) and reads containing adapters, in order to generate clean reads. HISAT2 (v2.1.0) was used to construct an index for the *B. aphidicola* reference genome (NZ_CP029204.1) obtained from the NCBI genome database (https://www.ncbi.nlm.nih.gov/datasets/genome/NZ_CP029204.1) (accessed on 16 March 2024). Paired-end clean reads were then aligned to this reference genome using HISAT2 (v2.1.0). After alignment, StringTie (v2.1.5) was employed to calculate the read count values mapped to each gene, which represented the raw gene expression levels. The differentially expressed genes (DEGs) were identified using the R package DESeq2 (v1.10.1) based on the obtained matrix of raw counts for each gene. Genes were considered differentially expressed between the two biotypes if adjusted *p* values were <0.05 and fold change values were >2. Based on raw counts, a heatmap of DEGs was visualized using an online tool (https://www.omicshare.com/tools/home/report/reportheatmap.html) (accessed on 28 March 2024).

### 2.5. Verification of Target Amino Acid Metabolic Pathways in Buchnera Mediated Biotype Differentiation of S. avenae

The results of transcriptome analysis showed that the metabolism of leucine (Leu), tryptophan (Trp), isoleucine (Ile), and valine (Val) may represent crucial amino acid metabolic pathways for the *Buchnera*-mediated biotype differentiation of *S. avenae*. In the subsequent study, these amino acids (Leu, Trp, Ile, or Val) were individually removed from the full-nutrition artificial diet, and the full-nutrition artificial diet was used as the control group to feed six biotypes separately. To be more specific, within 24 h, newborn nymphs randomly selected from the clonal complex of each biotype were transferred to a separate aphid feeder and raised under the conditions described above. All artificial diets were replaced every two days. Ten-day fecundities and the developmental time of nymphs of test aphid individuals were recorded. Each biotype was tested 20 times under each artificial diet.

### 2.6. Phylogenetic Analysis of Buchnera Among Six S. avenae Biotypes

For each clone of six *S. avenae* biotypes, five adult aphid individuals were collected, and DNA was extracted according to the method described above. To gather adequate data for a robust phylogenetic reconstruction, we amplified and sequenced fragments of six *Buchnera*-derived genes (i.e., *16S rDNA*, *Gnd*, *AtpD*, *LeuB*, *IlvD* and *TrpE*). *16S rDNA*, *Gnd*, and *AtpD* were used to detect the genetic differentiation of *Buchnera* in previous studies [34,35,36]. Based on the results of transcriptome analysis, *LeuB*, *IlvD* and *TrpE* were considered to be important genes for *Buchnera*-mediated *S. avenae* biotype differentiation. All primers are listed in Table S3. The PCR total reaction volume was 50 μL, containing 2 μL DNA template, 25 μL 2 × Es Taq MasterMix (Cwbio, Taizhou, China), 21 μL ddH_2_O and 1 μL for each primer. The PCR conditions were as follows: an initial denaturation at 94 °C for 5 min, followed by 35 cycles of 94 °C for 30 s, 45–60 °C for 30 s, and 72 °C for 1–2 min, and a final extension at 72 °C for 10 min. The annealing temperatures of each specific primer set were as follows: 45 °C for *AtpD*, 56 °C for *Gnd*, *LeuB*, *IlvD* and *TrpE*, and 60 °C for *16S rDNA*. PCR productions were gel-purified and then sequenced on an ABI 3730xl DNA Analyzer (Sangon, Shanghai, China).

Raw sequences were assembled in SeqMan II (DNAStar, Madison, WI, USA). Multiple alignments were performed with MEGA 5.0 and MAFFT 7.0 [37,38]. Gblocks 0.91b was used to remove unreliably aligned positions of *16S rDNA*, *Gnd*, *AtpD*, *LeuB*, *IlvD* and *TrpE* sequences [39]. The NJ phylogenetic tree was constructed for *Buchnera* in different biotypes of *S. avenae* using MEGA 5.0 with 1000 bootstrap replicates [37].

### 2.7. Statistical Analysis

A three-way ANOVA was used to evaluate the effects of ‘biotype’, ‘aphid stage’, and ‘host plant’, as well as the effects of their interactions on *Buchnera* abundance. Student’s *t*-tests further analyzed *Buchnera* abundance differences across aphid developmental stages. For the effects of ‘biotype’ and ‘host plant’, one-way ANOVA with Tukey’s test (α = 0.05) was conducted for further comparison. Two-way ANOVA was used to analyze the effects of ‘aphid biotype’ and ‘artificial diet’, as well as the effects of their interactions on all life-history traits of *S. avenae*. Further comparisons of ‘biotype’ and ‘artificial diet’ effects on life-history traits were performed using one-way ANOVA with Tukey’s test (α = 0.05). Differences between other data sets were also analyzed with one-way ANOVA followed by Tukey’s test (α = 0.05). When needed, data were log-transformed to satisfy the assumptions of normality and homoscedasticity required for these analyses. Principal component analysis (PCA) was conducted using the 10-day fecundity data of *S. avenae* on different wheat/barley varieties. Additionally, based on PCA, separate analyses were conducted for the 10 d fecundities and the total nymphal developmental time of different biotypes on artificial diets. Pearson correlations between 10 d fecundity and the abundance of *Buchnera* were assessed. All the above analyses were carried out in the software SPSS Statistics 23 (IBM, Armonk, NY, USA).

## 3. Results

### 3.1. Effects of Biotype, Host Plant, and Aphid Stage on Buchnera Abundance of S. avenae

Biotype, aphid stage, host plant and their interactions all showed significant effects (*p* < 0.001) on *Buchnera* abundance (Table S6). Biotype, aphid stage and host plant accounted for 23.16%, 47.69% and 12.12% of the total variance, respectively. Their interactions contributed little to the total variance (2.50–7.64%).

**Figure 1:**
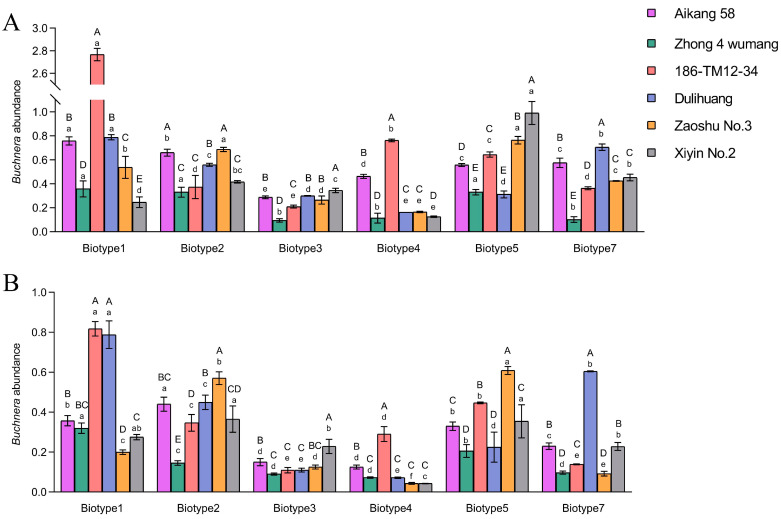
The *Buchnera* abundance in six *Sitobion avenae* biotypes ((**A**), 5-day-old nymphs; (**B**), adults) on different host plants. Error bars indicate ± SE; Different uppercase letters above the bars indicate significant differences in *Buchnera* abundance for the same biotype on different host plants, and lowercase letters indicate significant differences in *Buchnera* abundance among six biotypes on the same plant (α = 0.05, ANOVA followed by Tukey’s tests).

Significant differences (*F*_5,18_ = 29.60–1073.95, *p* < 0.001) in the abundance of *Buchnera* for *S. avenae* biotypes were found on different host plants (Figure 1). For instance, biotype 1’s 5-day-old nymphs exhibited significantly greater *Buchnera* abundance on the 186-TM12-34 variety compared to other varieties. When fed on different host plants, the *Buchnera* abundance and virulence response profiles for some biotypes showed a certain consistency. For example, the *Buchnera* abundance of 5-day-old nymphs of biotype 2 on Zaoshu No.3 (susceptible to biotype 2) was significantly higher than on other resistant varieties (i.e., Zhong 4 wumang, 186-TM12-34, Dulihuang, and Xiyin No.2; resistance to biotype 2). This phenomenon was also observed in biotype 2 adults, biotype 3 nymphs, biotype 4 nymphs and adults, biotype 5 nymphs, and biotype 7 nymphs and adults.

*Buchnera* abundance also differed significantly between biotypes when fed on the same variety (*F*_5,18_ = 25.34–1682.87, *p* < 0.001). For example, when fed on Aikang 58 and 186-TM12-34, the abundance of *Buchnera* in biotype 1 was significantly higher than that in other biotypes (Figure 1A). In addition, combining the data on all host plants, 5-day-old nymphs had a significantly higher abundance of *Buchnera* than adults for all biotypes (*t* = 2.42–5.75, df = 46, *p* < 0.05; Table S7).

### 3.2. Differences in the Virulence Response Profiles of Six S. avenae Biotypes on Wheat/Barley Varieties Before and After Rifampin Treatments

After being fed on diets with 2 μg/mL rifampicin, *Buchnera* abundance decreased by about 67.7% compared with the control, and 10 d fecundity decreased to 43.3% of the control (Figure S2). This indicated that under the treatment of this concentration, the abundance of *Buchnera* decreased significantly, and the aphids had a certain fecundity. Therefore, rifampicin with a concentration of 2 μg/mL was selected to be added to artificial diets for subsequent antibiotic treatment experiments. Subsequent verification results (Figure S3) showed that for all biotypes, the abundance of *Buchnera* was detected to be significantly reduced under rifampicin treatments at this concentration (*t* = 17.29–41.71, df = 6, *p* < 0.001).

**Figure 2:**
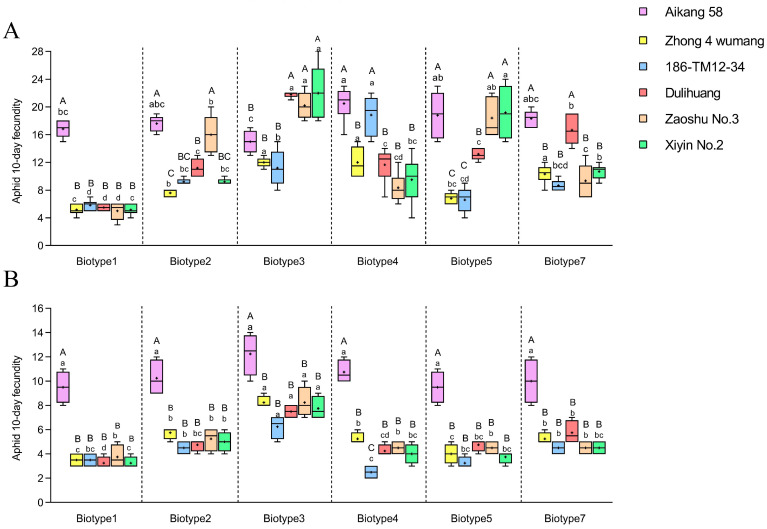
Comparisons of 10 d fecundity for six *Sitobion avenae* biotypes on different varieties ((**A**), before the rifampicin treatment; (**B**), after the rifampicin treatment). Error bars indicate ± SE; Different uppercase letters above the bars indicate significant differences in the fecundity for the same biotype on different host plants, and lowercase letters indicate significant differences in fecundity among six biotypes on the same plant (α = 0.05, ANOVA followed by Tukey’s tests).

The resistance performance of the six biotypes on each wheat/barley variety before rifampicin treatments was consistent with the previous results (Table 1 and Table S1), which verified the original virulence response profile of the six biotypes selected in this study. Furthermore, each biotype showed lower 10 d fecundities on host varieties that exhibited a certain level of resistance (*F*_5,30_ = 22.58–160.41, *p* < 0.001; Figure 2A). After rifampicin treatment, except for biotype 1, the virulence profiles of the other five biotypes of *S. avenae* changed, and they lost the ability to overcome the resistance of some varieties. For example, biotype 2 lost its virulence to Zaoshu No.3, and biotype 3 lost the ability to overcome the resistance of Dulihuang, Zaoshu No.3 and Xiyin No.2. Overall, the virulence response profiles among the six biotypes of *S. avenae* became similar after antibiotic treatments. For each biotype, 10 d fecundities on the five resistant host varieties (i.e., Zhong 4 wumang, 186-TM12-34, Dulihuang, Zaoshu No.3, and Xiyin No.2) were significantly lower than those on Aikang 58 (*F*_5,18_ = 14.38–71.44, *p* < 0.001). Pearson correlation analysis showed significant positive correlations between aphid fecundity and *Buchnera* abundance in biotypes 1, 2, and 4 (Table S8; *r* = 0.889–0.967, *p* < 0.05).

Before rifampin treatment, PCA of the 10-day fecundities on the six wheat/barley varieties revealed that the first two principal components (PC1 and PC2) accounted for 75.11% of the overall variation (Table S9; 46.30% and 28.82% for PC1 and PC2, respectively). After the rifampin treatment, PC1 and PC2 explained 87.10% of the total variation (Table S9; 73.76% and 13.33% for PC1 and PC2, respectively). Before rifampin treatment, the contribution of the five resistant varieties to PC1 varied greatly (0.319–0.884), while with rifampin antibiotic treatment, the differences in their contribution to PC1 were smaller (0.805–0.944).

### 3.3. Identification of Differentially Expressed Genes in Buchnera Between Two S. avenae Biotypes

**Figure 3:**
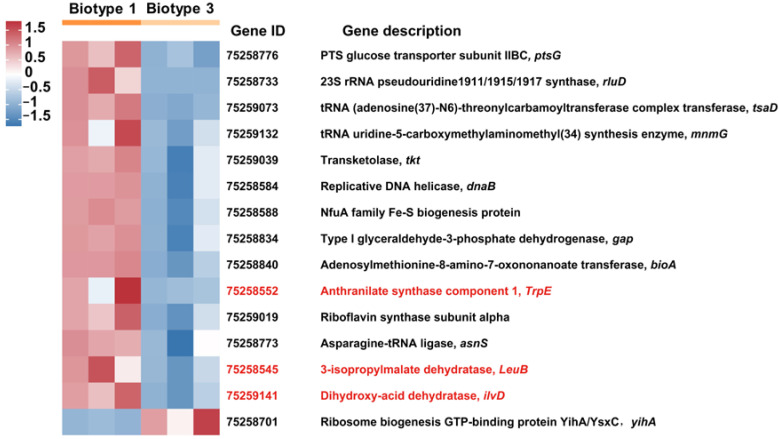
A heatmap of DEGs of *Buchnera* between two *Sitobion avenae* biotypes (genes with expression higher and lower than the mean are indicated by red and blue, respectively).

A total of 15 differentially expressed genes (DEGs; adjusted *p* values < 0.05, fold change values > 2) of *Buchnera* were detected between *S. avenae* biotypes 1 and 3 (Figure 3). In biotype 1, fourteen genes showed significant up-regulation compared to biotype 3, with log2(fold change) values ranging from 1.05 to 3.01, and one gene was down-regulated (log2(fold change) values being 1.18). Among them, three DEGs (i.e., 3-isopropylmalate dehydratase (*LeuB*, 75258545); dihydroxy-acid dehydratase (*IlvD*, 75259141); and anthranilate synthase component 1 (*TrpE*, 75258552)) were EAAs synthesis-related genes (i.e., *LeuB* related to Leu; *TrpE* related to Trp; *IlvD* related to Leu, Ile, and Val).

### 3.4. The Developmental Times of Nymphs and the Fecundity for S. avenae Biotypes Fed on Different Artificial Diets

**Figure 4:**
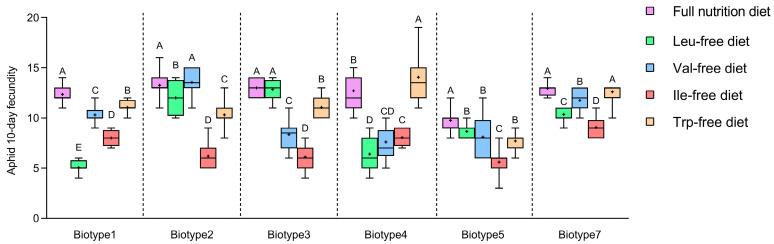
Comparisons of 10 d fecundity for six *Sitobion avenae* biotypes on different artificial diets. Error bars indicate ± SE; Different uppercase letters above the bars indicate significant differences in the fecundity for the same biotype on different artificial diets, and lowercase letters indicate significant differences in fecundity among six biotypes on the same artificial diet (α = 0.05, ANOVA followed by Tukey’s tests).

The developmental times of nymphs and the 10-day fecundity of *S. avenae* were significantly influenced by artificial diets, biotypes, and their interaction (Table S10). Artificial diets and biotypes account for 22.69–66.44% and 22.00–67.13% of the total variance of each trait, respectively, and their interaction explained 4.64–11.36%. For each *S. avenae* biotype, significant differences were found in the 10 d fecundity of *S. avenae* clones after being fed on different artificial diets (*F*_4,95_ = 30.99–287.74, *p* < 0.001) (Figure 4). For example, biotypes 1 and 5 had significantly higher 10 d fecundity on the full-nutrient artificial diet compared to the nutrient-deficient artificial diets (i.e., Leu-free, Val-free, Ile-free, and Trp-free). Compared with the full-nutrient artificial diet, biotypes 2 and 3 showed similar fecundity on artificial diets lacking Val and Leu, respectively.

Significant differences were also found in the developmental time of first- to fourth-instar nymphs and the total developmental time of nymphs for each biotype fed with different artificial diets (Table 2). For biotypes 1, 4 and 7, the lack of any EAAs significantly prolonged their total nymphal developmental time, whereas for biotype 3, the total developmental time of nymphs remained unaffected by the missing amino acids in the artificial diets. The lack of Ile and Val significantly affected the total developmental time of nymphs of biotypes 2 and 5, respectively. The total developmental time of nymphs also differed significantly between biotypes when fed on the same artificial diet (Table 2). For example, when fed on the Leu-free artificial diet, the total nymphal developmental time of biotypes 1, 4, and 5 was significantly longer than that of other biotypes. The PCA of total nymphal developmental time on artificial diets revealed that the first two components (PC1 and PC2) accounted for 59.87% of the overall variation (Table 3; 41.90% and 17.97% for PC1 and PC2, respectively). In addition, PC1 was associated mainly with Leu. For 10 d fecundity PCA analysis, PC1 and PC2 explained 67.61% of the total variation (Table 3; 38.31% and 29.30% for PC1 and PC2, respectively), and Trp contributed the most to PC1. The high correlations of Leu and Trp with the development and reproduction traits of six biotypes suggested that they might be the key amino acids for the biotype differentiation of *S. avenae*.

### 3.5. Phylogenetic Relationships Among S. avenae Biotypes Based on the Gene Datasets of Buchnera

**Figure 5:**
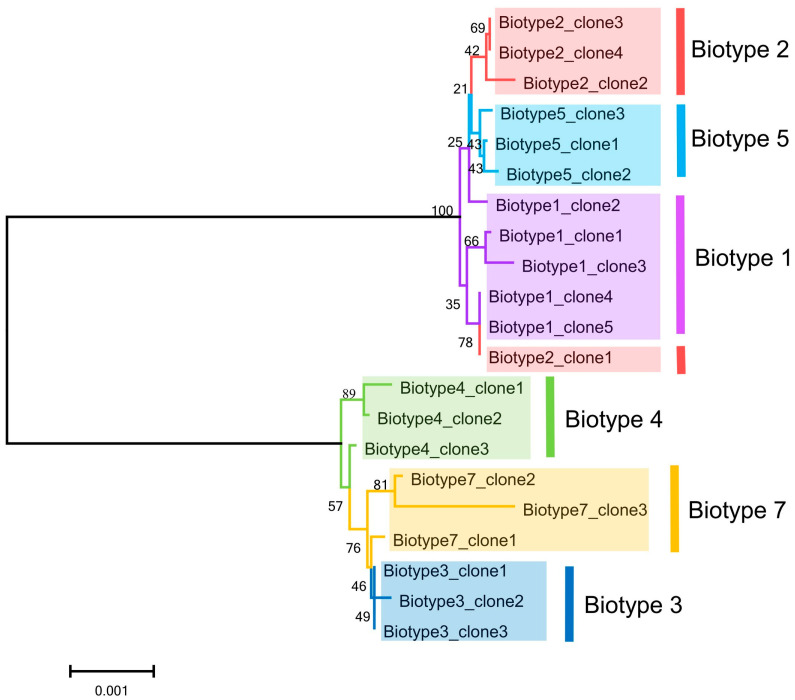
A neighbor-joining dendrogram based on the combined *16S rDNA*, *Gnd*, *AtpD*, *LeuB*, *IlvD*, *TrpE* datasets of *Buchnera* from six *Sitobion avenae* biotypes.

Except for *16S rDNA*, the other five gene fragments were characterized by high AT content (*Gnd*, 74.4%; *AtpD*, 64.2%; *LeuB*, 70.1%; *IlvD*, 70.7%; *TrpE*, 71.5%; Table S11). For EAA-related genes (i.e., *LeuB*, *IlvD*, and *TrpE*), *LeuB* and *TrpE* were rich in variable (*LeuB*, 2.07%; *TrpE*, 1.60%) and parsimony informative sites (*LeuB*, 2.07%; *TrpE*, 1.60%). An NJ (neighbor-joining) phylogenetic tree based on the combined gene datasets was created to show the genetic relationships of *Buchnera* from six *S. avenae* biotypes (Figure 5). All samples formed two major branches, and all clones of biotypes 1, 2, and 5 clustered together, forming one clade. Another major clade included clones of biotypes 3, 4, and 7. Furthermore, all clones of biotypes 5 or 3 were clustered together, forming minor clades, respectively. For each of the other biotypes, *Buchnera* was also relatively closely genetically related among aphid clones, with most of the clones clustered together. The above results showed that *Buchnera* sequences in different clones of the same *S. avenae* biotype had little difference, and their genetic relationships were relatively close.

## 4. Discussion

Aphid populations are well-documented for their tendency towards rapid biotype differentiation [3]. As an important primary symbiont in aphids, *Buchnera* can play a critical role in the host adaptation of aphids by synthesizing EAAs [11]. However, the involvement of *Buchnera* in aphid biotype differentiation and the mechanisms have been less studied. In this research, the English grain aphid (*S. avenae*) served as a model organism to investigate these concerns. Previous studies, including ours, have identified multiple biotypes of this aphid [26,40]. The results of this study showed significant differences in the abundance of *Buchnera* among *S. avenae* biotypes (Figure 1 and Table S6). Aphids can obtain adequate nutrients by altering *Buchnera* abundance in response to deficient EAAs in resistant hosts [41,42]. The variability in the abundance of *Buchnera* among pea aphid *A. pisum* biotypes was found in a previous study [17]. The research results of Zhang et al. [18] on the abundance of *Buchnera* in *Aphis gossypii* (Glover) biotypes also showed that the biotype on cucumber contained more *Buchnera* than the biotype on cotton, and the higher abundance of *Buchnera* can provide more essential amino acids. The composition and contents of free amino acids in the phloem of different Triticeae crops are varied, and the less EAAs in resistant Triticeae crop varieties may be an important factor in aphid resistance [27]. Therefore, we speculate that the distinction in the virulence of *S. avenae* biotypes on resistant wheat/barley varieties may be related to the differences in the abundance of *Buchnera* in each biotype. Interestingly, in most cases, each biotype had a lower abundance of *Buchnera* on the resistant wheat/barley varieties than on the susceptible varieties. For example, both 5-day-old nymphs and adults of biotype 4 had a significantly higher abundance of *Buchnera* on 186-TM12-34 than other varieties. Furthermore, for all biotypes, we detected differences in the abundance of *Buchnera* in different stages of aphids. Specifically, the abundance of *Buchnera* in 5-day-old nymphs was significantly higher than that in adults (Table S7). This is consistent with a previous study showing that the stage of the aphid can affect the abundance of *Buchnera* [43]. This may be related to the fact that aphids require *Buchnera* to help to provide large nutrient reserves for growth and development, prior to eclosion. In this study, we also detected significant correlations between aphid fecundity and *Buchnera* abundance (Table S8).

To further clarify the role of *Buchnera* in the biotype divergence of *S. avenae*, we treated six biotypes with the antibiotic rifampicin to reduce the abundance of *Buchnera* and detected changes in their virulence response profiles on the five wheat/barley varieties. After antibiotic treatments, five biotypes (i.e., biotype 2–5 and 7) altered their unique virulence profiles on the wheat/barley cultivars, and the resistance performance on each host plant became similar to biotype 1. For example, biotype 2 lost its virulence to Zaoshu No.3, and biotype 3 lost the ability to overcome the resistance of Dulihuang, Zaoshu No.3 and Xiyin No.2. Changes in the virulence response profiles of these biotypes following antibiotic treatments were also verified by testing their life-history traits (i.e., 10 d fecundity) on these wheat/barley varieties. That is, after rifampicin treatments, the 10 d fecundity of each biotype on the susceptible control variety Aikang 58 was significantly higher than that on all five wheat/barley varieties (i.e., Zhong 4 wumang, 186-TM12-34, Dulihuang, Zaoshu No.3, and Xiyin No.2).

Furthermore, the results of principal component analysis (PCA) also indicate that, compared with before antibiotic treatment, after antibiotic treatment, the contributions of the five resistant varieties to the fecundity of *S. avenae* became more similar (Table S9). A reasonable explanation is that after the abundance of *Buchnera* was reduced, the ability of each biotype to synthesize EAAs was reduced, leading to the consistent performance of different biotypes on these resistant wheat/barley varieties. We also found that compared with the five resistant varieties, the 10 d fecundity of each biotype on the susceptible variety Aikang 58 was at a higher level. Susceptible Triticeae crop varieties can provide more sufficient EAAs for aphids [27]. EAAs contained in the phloem of Aikang 58 may be relatively sufficient for *S. avenae*, and the lower abundance of *Buchnera* can meet the needs of aphids’ growth and reproduction on this variety, but this needs to be further explored. Luna et al. [44] also used antibiotics to reduce the abundance of *Buchnera* in *D. noxia* biotype RWA2, and the virulence of this biotype on the wheat variety TAM 107 was weakened after treatments. This is consistent with the research findings of this study, and these results can provide evidence for *Buchnera*’s involvement in the differentiation of aphid biotypes. Rifampicin is usually selected to reduce the abundance of *Buchnera*. Based on preliminary experiments (Figure S2) and subsequent validation assays (Figure S3), we established the conditions of 2 μg/mL for 24 h for the rifampicin treatment. It is worth noting that in other studies [31,45], rifampicin was used at 50 μg/mL for 48 h to completely eliminate *Buchnera*. However, in this study, the goal of the rifampicin treatment was to reduce the abundance of *Buchnera* while allowing aphids to retain certain reproductive capabilities. If the concentration of rifampicin exceeds 2 μg/mL, the reproductive capacity of *S. avenae* cannot meet the requirements for subsequent virulence assays.

In order to identify the key genes and EAAs participating in the biotype differentiation of *S. avenae* mediated by *Buchnera*, the differentially expressed genes (DEGs) of *Buchnera* between biotypes 1 and 3 were detected by transcriptome analyses. Three DEGs encoding for anthranilate synthase component 1, 3-isopropylmalate dehydratase, and dihydroxy-acid dehydratase were found (Figure 3), involved in the synthesis of Trp, Leu, Ile, and Val [46,47]. These genes and EAA metabolism may contribute to the biotype differentiation of *S. avenae*. Based on the transcriptome analysis, one Leu metabolism-related gene of *Buchnera* in *D. noxia* biotype RWA2 was significantly up-regulated compared with the biotype RWA1. Therefore, the Leu metabolism of *Buchnera* was thought to have a critical role in biotype differentiation for this aphid [20]. In another study [17], Arg metabolism was considered to be an important metabolic pathway involved in the differentiation of *A. pisum* biotypes mediated by *Buchnera*, and the alanine aminotransferase differentially expressed in different biotypes of this aphid was the key to regulating Arg metabolism in *Buchnera*. For different species of aphids, the differences in EAA metabolism involved in the differentiation of biotypes mediated by *Buchnera* may be related to the different compositions of free EAAs in their host plants. Another reasonable explanation is that certain genetic variations of *Buchnera* exist among different species of aphids [21,22].

For the purpose of further clarifying the key EAA metabolism of *Buchnera* involved in the biotype differentiation of *S. avenae*, we removed the above-mentioned amino acids (i.e., Leu, Trp, Ile, and Val) from the full-nutrition artificial diet and compared the life-history traits of different biotypes fed on different artificial diets. The results of one-way ANOVA (Table 2) indicated that the life-history traits of different biotypes were varied on the above artificial diets. For example, compared with the full-nutrient artificial diet, the total developmental time of nymphs was significantly prolonged for *S. avenae* biotypes 1, 4, 5, and 7 fed on the Trp-free artificial diet. This is consistent with the research of Chen et al. [48]. However, for biotypes 2 and 3, a lack of Trp had little effect on their development. In a previous study, Val and Leu in artificial diets were considered dispensable for the reproduction of *S. avenae* [47], consistent with our results of 10 d fecundity of biotypes 2 and 3 in this study. However, for other biotypes, the role of these two amino acids in the reproduction of *S. avenae* was critical in this study. Different aphid biotypes may have different requirements for different EAAs in food, so it is also necessary to select as many biotypes or genotypes as possible for tests when configuring artificial diets for a certain aphid. Three-way ANOVA results showed that “biotype”, “artificial diet” and their interaction significantly affected the developmental time of nymphs and the fecundity of aphids (Table S10). Different biotypes of aphids showing variation in their fitness performance on artificial diets deficient in EAAs were also found in *A. gossypii* [28]. The above research results all indicate that different aphid biotypes have varying dependencies on essential amino acids (EAAs), which may lead to their specific virulence responses to host plants with different EAA compositions, and *Buchnera* in host aphids may mediate this process [27,28]. The study by Chung et al. [49] found significant associations between molecular variation in *Buchnera* and the amino acid requirements of the pea aphid *A. pisum*. In this study, a certain genetic differentiation of *Buchnera* was also detected between different *S. avenae* biotypes with different EAA requirements (Figure 5). This can supply additional evidence for the involvement of *Buchnera* in the biotype differentiation of *S. avenae* from another perspective and also provide new evidence for the parallel evolution hypothesis between aphids and *Buchnera* proposed in previous studies [22,50].

Principal component analysis (PCA) results (Table 3) showed that the artificial diets deficient in Leu and Trp had the greatest association with the total development duration of nymphs and aphid fecundity, respectively, suggesting that these two EAA metabolic pathways may play a critical role in the biotype differentiation of *S. avenae* mediated by *Buchnera*. In a previous study [16], molecular sequencing was used to detect mutations of *Buchnera* genes in different aphid biotypes to clarify the important genes of EAA metabolism involved in biotype differentiation. Interestingly, based on our sequencing analyses, high mutation rates were also detected at the *LeuB* and *TrpE* sites for different *S. avenae* biotypes in this research. All of the above suggests a potential role for the metabolism of two essential amino acids (i.e., Leu and Trp) and the associated synthesis genes (i.e., *LeuB* and *TrpE*) in the biotype differentiation of *S. avenae* mediated by *Buchnera*. In the future breeding of aphid-resistant varieties, the content of these two free amino acids in the phloem of plants should be considered.

Based on the above-mentioned results, we believe that this study suggests a potential role of *Buchnera* in *S. avenae’s* damage to host plants. Other studies indicate that horizontally transferred genes *amiD* and *ldcA1*, also detected in the bacteriocytes of *S. avenae*, can protect *Buchnera* from attacks by the host immune system [51,52]. Using the dsRNA of these two genes to reduce the abundance of *Buchnera*, through specific expression in transgenic plants or artificial spraying, may be a promising method for controlling *S. avenae* in the future. Aphids have multiple active regulatory mechanisms in the abundance and amino acid metabolism of *Buchnera* [13,51], but the variation in the regulation ability of different *S. avenae* biotypes is still unclear, as is their role in the differentiation of aphid biotypes, which needs to be the focus of future research.

## 5. Conclusions

In summary, this study clarified the potential role of *Buchnera* in the biotype differentiation of *S. avenae* through *Buchnera* abundance determination and antibiotic treatments. Transcriptome sequencing identified candidate genes involved in the metabolism of essential amino acids (EAAs) in *Buchnera* that are related to *S. avenae* biotype differentiation. Multi-locus molecular sequencing and life-history trait bioassays for aphids on various artificial diets further clarified the important roles of EAA (i.e., Leu and Trp) metabolic pathways in *Buchnera*-mediated *S. avenae* biotype differentiation. Our research is significant for elucidating the mechanism of biotype differentiation in *S. avenae* and deepening the understanding of *Buchnera*’s potential functions in aphid–plant interactions. These insights can provide new perspectives for developing more targeted and effective aphid control strategies, improving pest management practices and reducing reliance on chemical insecticides.

## Figures and Tables

**Table 1:** Response of five wheat/barley varieties to six *Sitobion avenae* biotypes before and after the rifampin treatment.

Biotypes	Wheat/Barley Varieties				
	Zhong 4 Wumang	186-TM12-34	Dulihuang	Zaoshu No.3	Xiyin No.2
Biotype 1	R 0.47 ^a^ (R 0.42) ^b^	R 0.53 (R 0.50)	R 0.44 (R 0.58)	R 0.50 (R 0.67)	R 0.47 (R 0.58)
Biotype 2	R 0.41 (R 0.52)	R 0.52 (R 0.38)	R 0.65 (R 0.41)	S 1.02 (R 0.45)	R 0.52 (R 0.48)
Biotype 3	R 0.82 (R 0.70)	R 0.78 (R 0.53)	S 1.69 (R 0.67)	S 1.76 (R 0.77)	S 1.73 (R 0.70)
Biotype 4	R 0.60 (R 0.63)	S 1.01 (R 0.32)	R 0.54 (R 0.58)	R 0.38 (R 0.53)	R 0.45 (R 0.42)
Biotype 5	R 0.30 (R 0.32)	R 0.32 (R 0.29)	R 0.73 (R 0.43)	S 1.03 (R 0.39)	S 1.13 (R 0.32)
Biotype 7	R 0.51 (R 0.36)	R 0.43 (R 0.33)	S 1.02 (R 0.39)	R 0.42 (R 0.33)	R 0.52 (R 0.27)

Note: R, resistant; S, susceptible; The value represents the ratio of aphid counts (>0.9 indicates susceptivity and the opposite indicates resistance); ^a^ represents before the rifampin treatment, ^b^ represents after the rifampin treatment.

**Table 2:** Comparisons of the developmental times of nymphs (mean ± SE) for six *Sitobion avenae* biotypes on different artificial diets.

Instar Duration of Nymphs	Artificial Diets	Biotype 1	Biotype 2	Biotype 3	Biotype 4	Biotype 5	Biotype 7	*F*_5,114_	*p*
DT1 (d)	Full nutrition	2.40 ± 0.11Aab	2.20 ± 0.09 ABb	2.35 ± 0.11 ABab	2.65 ± 0.11 Ca	2.30 ± 0.11 Aab	2.10 ± 0.07 Cb	3.520	0.005
	Leu-free	2.45 ± 0.11 Ab	2.40 ± 0.11 Ab	2.50 ± 0.11 Ab	3.55 ± 0.11 Aa	2.20 ± 0.09 Ab	2.50 ± 0.11 ABb	18.714	<0.001
	Val-free	2.50 ± 0.11 Aa	2.00 ± 0.00 Bc	2.00 ± 0.00 Bc	2.45 ± 0.11 Cab	2.40 ± 0.11 Aab	2.15 ± 0.08 BCbc	6.853	<0.001
	Ile-free	2.70 ± 0.11 Aa	2.35 ± 0.11 Aabc	2.25 ± 0.10 ABbc	2.60 ± 0.511 Cab	2.10 ± 0.12 Ac	2.25 ± 0.10 ABCbc	4.479	<0.001
	Trp-free	2.55 ± 0.11 Ab	2.40 ± 0.11 Ab	2.55 ± 0.11 Ab	3.15 ± 0.08 Ba	2.25 ± 0.16 Ab	2.55 ± 0.11 Ab	6.690	<0.001
	*F*_4,95_	1.053	3.231	5.023	18.259	0.856	4.395	/	/
	*p*	0.384	0.016	0.001	<0.001	0.494	0.003	/	/
DT2 (d)	Full nutrition	2.20 ± 0.12 Ba	1.90 ± 0.12 BCa	2.25 ± 0.10 ABa	2.30 ± 0.13 Ca	2.00 ± 0.10 Ca	2.05 ± 0.05 Ba	2.171	0.062
	Leu-free	2.95 ± 0.11 Aa	2.15 ± 0.08 Bb	2.15 ± 0.08 ABb	2.75 ± 0.14 ABa	2.60 ± 0.11 ABa	2.15 ± 0.08 Bb	11.460	<0.001
	Val-free	3.05 ± 0.14 Aa	2.55 ± 0.11 Aab	2.45 ± 0.15 Ab	2.85 ± 0.13 ABab	2.90 ± 0.12 Aab	2.55 ± 0.11 Aab	3.451	0.006
	Ile-free	2.65 ± 0.11 Aa	1.60 ± 0.11 Cc	2.25 ± 0.10 ABab	2.55 ± 0.11 BCab	2.40 ± 0.11 BCab	2.20 ± 0.10 Bb	12.144	<0.001
	Trp-free	2.70 ± 0.11 Ab	1.85 ± 0.08 BCe	2.00 ± 0.00 Bde	3.00 ± 0.00 Aa	2.40 ± 0.11 BCc	2.05 ± 0.08 Bcd	31.047	<0.001
	*F*_4,95_	8.030	11.892	2.700	5.543	8.479	4.951	/	/
	*p*	<0.001	<0.001	0.035	<0.001	<0.001	0.001	/	/
DT3 (d)	Full nutrition	2.40 ± 0.11 Bab	2.30 ± 0.11 ABab	2.15 ± 0.08 Bab	2.40 ± 0.11 Cab	2.55 ± 0.14 Ba	2.05 ± 0.05 Bb	3.142	0.011
	Leu-free	3.20 ± 0.12 Aa	2.60 ± 0.11 Abc	2.30 ± 0.11 ABbc	2.65 ± 0.22 BCbc	2.80 ± 0.09 ABab	2.15 ± 0.08 Bc	8.212	<0.001
	Val-free	3.30 ± 0.11 Aa	2.20 ± 0.09 ABc	2.65 ± 0.15 Ab	3.35 ± 0.11 Aa	3.10 ± 0.07 Aa	2.55 ± 0.11 Abc	17.857	<0.001
	Ile-free	2.65 ± 0.11 Ba	2.00 ± 0.00 Bc	2.50 ± 0.20 ABab	2.70 ± 0.11 BCa	2.55 ± 0.11 Bab	2.15 ± 0.11 Bbc	5.537	<0.001
	Trp-free	2.25 ± 0.12 Bb	2.55 ± 0.17 Ab	2.10 ± 0.07 Bb	3.15 ± 0.21 ABa	2.50 ± 0.14 Bb	2.05 ± 0.05 Bb	8.731	<0.001
	*F*_4, 95_	17.197	5.089	3.211	5.907	5.068	5.857	/	/
	*p*	<0.001	<0.001	0.016	<0.001	<0.001	<0.001	/	/
DT4 (d)	Full nutrition	2.60 ± 0.11 Bb	3.50 ± 0.14 ABa	2.60 ± 0.11 Bb	2.35 ± 0.15 Cb	3.80 ± 0.14 Aa	2.25 ± 0.10 Cb	26.021	<0.001
	Leu-free	3.40 ± 0.15 Aab	3.15 ± 0.15 BCbc	3.20 ± 0.16 Abc	3.10 ± 0.22 ABbc	3.85 ± 0.13 Aa	2.60 ± 0.11 Bc	6.822	<0.001
	Val-free	3.20 ± 0.09 Aab	3.00 ± 0.10 Cbc	2.75 ± 0.12 ABc	3.10 ± 0.07 ABabc	3.50 ± 0.15 Aa	3.10 ± 0.07 Aabc	5.391	<0.001
	Ile-free	2.75 ± 0.09 Bb	3.00 ± 0.13 Cab	2.90 ± 0.16 ABab	2.70 ± 0.11 BCb	3.40 ± 0.20 Aa	2.70 ± 0.11 Bb	3.862	0.003
	Trp-free	2.75 ± 0.12 Bb	3.70 ± 0.11 Aa	2.90 ± 0.07 ABb	3.50 ± 0.17 Aa	4.00 ± 0.22 Aa	2.90 ± 0.07 ABb	14.061	<0.001
	*F*_4,95_	8.434	6.355	3.000	8.437	2.155	11.969	/	/
	*p*	<0.001	<0.001	0.022	<0.001	0.080	<0.001	/	/
DT (d)	Full nutrition	9.60 ± 0.15 Cb	9.90 ± 0.18 ABb	9.35 ± 0.24 Ab	9.70 ± 0.18 Db	10.65 ± 0.21 BCa	8.45 ± 0.11 Cc	15.351	<0.001
	Leu-free	12.00 ± 0.25 Aa	10.30 ± 0.16 ABb	10.15 ± 0.15 Ab	12.05 ± 0.29 Ba	11.45 ± 0.14ABa	9.40 ± 0.15 Bc	29.953	<0.001
	Val-free	12.05 ± 0.21 Aa	9.75 ± 0.18 Bb	9.85 ± 0.25 Ab	11.75 ± 0.18 Ba	11.90 ± 0.26 Aa	10.35 ± 0.11 Ab	27.556	<0.001
	Ile-free	10.75 ± 0.18 Ba	8.95 ± 0.17 Cd	9.90 ± 0.23 Abc	10.55 ± 0.21 Cab	10.45 ± 0.23 Cab	9.30 ± 0.18 Bcd	13.140	<0.001
	Trp-free	10.25 ± 0.19 Bc	10.50 ± 0.15 Ac	9.55 ± 0.11 Ad	12.80 ± 0.09 Aa	11.15 ± 0.24 ABCb	9.65 ± 0.11 Bd	57.118	<0.001
	*F*_4,95_	29.434	12.764	2.316	37.686	7.105	25.383	/	/
	*p*	<0.001	<0.001	0.063	<0.001	<0.001	<0.001	/	/

Note: DT1 to DT4, represent the developmental time of first- to fourth-instar nymphs; DT, represents the total developmental time of nymphs; Different letters within a column (uppercase letters) and a row (lowercase letters) indicate significant differences among artificial diets and biotypes (α = 0.05, ANOVA followed by Tukey’s tests), respectively.

**Table 3:** Principal component analysis (PCA) of the total developmental time of nymph and 10 d fecundity for six *Sitobion avenae* biotypes on artificial diets.

Diet Types	The Total Developmental Time of Nymph			The 10-Day Fecundity		
	PC1	PC2	PC3	PC1	PC2	PC3
	Correlation with principal components					
Full nutrition	0.483	0.718	0.467	0.711	0.485	−0.270
Leu-free	0.801	−0.100	−0.018	−0.179	0.837	−0.309
Val-free	0.665	−0.128	−0.389	0.167	0.659	0.707
Ile-free	0.684	0.194	−0.382	0.776	−0.296	0.338
Trp-free	0.556	−0.565	0.555	0.865	−0.087	−0.282
	Variance explained in PCA (%)					
Percentage of principal components (%)	41.90	17.97	16.48	38.31	29.30	17.26
Cumulative percentage (%)	41.90	59.87	76.35	38.31	67.61	84.86

## Data Availability

The original RNA-seq data have been uploaded to the NCBI_SRA database (PRJNA575173).

## Supplementary Materials

The following supporting information can be downloaded at: https://www.mdpi.com/article/10.3390/insects15120980/s1, Table S1: The specific virulence profiles of the six *Sitobion avenae* biotypes selected for this study on resistant wheat/barley varieties; Table S2: Sample collection and genetic information for *Sitobion avenae* biotypes; Table S3: The sequences of primers used in this study; Table S4: Facultative endosymbionts examined in this study; Table S5: Formula of the full-nutrition artificial diet for *Sitobion avenae*; Table S6: Three-way analyses of variance for *Buchnera* abundance affected by host plant, biotype and developmental stage of *Sitobion avenae*; Table S7: Comparisons of *Buchnera* abundance in 5-day-old nymphs and adults for six *Sitobion avenae* biotypes; Table S8: Pearson correlation analyses between 10 d fecundity and *Buchnera* abundance for six biotypes of *Sitobion avenae* (5-day-old nymphs and adults); Table S9: Principal component analyses (PCA) of 10 d fecundity for six biotypes of *Sitobion avenae* on six varieties before and after rifampin treatments; Table S10: Two-way analyses of variance for the developmental times of nymphs and 10 d fecundity for *Sitobion avenae* affected by artificial diets and biotypes; Table S11: Characters of DNA fragments used in phylogenetic analyses; Figure S1: Electrophoretic detection of the facultative endosymbiosis for clone 3 of six *Sitobion avenae* biotypes; Figure S2: The differences in *Buchnera* abundance and 10 d fecundity of *Sitobion avenae* under rifampicin treatments; Figure S3: Changes in the abundance of *Buchnera* of six *Sitobion avenae* biotypes under rifampicin treatments.
