# Supplementary material for: Potential Involvement of Buchnera aphidicola (Enterobacteriales, Enterobacteriaceae) in Biotype Differentiation of Sitobion avenae (Hemiptera: Aphididae)
Source: Insects. 2024 Dec 11;15(12):980. doi: 10.3390/insects15120980 (PMC11679945; doi:10.3390/insects15120980)
Supplement: Supplementary file 1 [file insects-15-00980-s001.zip › insects-3307036-supplementary.pdf]

Table S1. The specific virulence profiles of the six *Sitobion avenae* biotypes selected for this study on resistant wheat/barley varieties.

**Biotypes**	**Wheat/barley varieties**					**Reference**
	**Zhong 4 wumang**	**186-TM12-34**	**Dulihuang**	**Zaoshu No.3**	**Xiyin No.2**	
Biotype 1	R	R	R	R	R	Wang et al. 2020b
Biotype 2	R	R	R	S	R	Wang et al. 2020b
Biotype 3	R	R	S	S	S	Wang et al. 2020b
Biotype 4	R	S	R	R	R	Wang et al. 2020b
Biotype 5	R	R	R	S	S	Wang et al. 2020b
Biotype 7	R	R	S	R	R	Unpublished

Note: R, resistant; S, susceptible; Wheat varieties: Zhong 4 wumang and 186-TM12-34; Barly varieties: Dulihuang, Zaoshu No.3, and Xiyin No.2.

Table S2. Sample collection and genetic information for *Sitobion avenae* biotypes.

Biotypes	Clones	Host	Sampling location	GPS Coordinates	Collection Date	Six microsatellite locus											
						Sm10		S5.L		Sa4Σ		Sm17b		Sm17		Sm12	
Biotypes 1	1	Wheat	Ganzhou District, Zhangye City, Gansu Province	100°28′ E; 38°54′ N	2016.04	154	166	227	227	165	167	204	204	94	94	149	156
Biotypes 1	2	Wheat	Minle County, Zhangye City, Gansu Province	100°46′ E; 38°25′ N	2016.04	154	156	223	223	152	152	204	204	94	94	128	147
Biotypes 1	3	Wheat	Gaotai County, Zhangye City, Gansu Province	99°41′ E; 39°25′ N	2016.07	154	164	212	227	157	157	204	204	94	94	135	156
Biotypes 1	4	Wheat	Yangling District, Yangling City, Shaanxi Province	108°03′E; 34°15′N	2016.04	154	164	214	223	163	165	196	196	94	98	159	159
Biotypes 1	5	Wheat	Biyang County, Zhumadian City, Henan Province	113°20′ E; 32°46′ N	2016.04	156	156	214	227	163	165	196	196	98	100	139	155
Biotypes 2	1	Barley	Haiyan County, Haibei City, Qinghai Province	100°51′E; 37°01′N	2016.07	156	164	223	227	163	163	196	196	94	98	156	156
Biotypes 2	2	Barley	Minle County, Zhangye City, Gansu Province	100°46′ E; 38°25′ N	2016.04	154	162	216	227	167	167	206	206	94	100	139	139
Biotypes 2	3	Wheat	Yangling District, Yangling City, Shaanxi Province	108°03′E; 34°15′N	2016.04	154	164	217	223	157	163	194	196	96	104	150	157
Biotypes 2	4	Wheat	Lianchi District, Baoding City, Hebei Province	115°27′ E; 38°50′ N	2018.04	164	164	223	226	163	163	196	196	94	100	135	158
Biotypes 3	1	Barley	Biyang County, Zhumadian City, Henan Province	113°20′ E; 32°46′ N	2016.04	154	154	215	230	163	163	194	196	100	100	135	149
Biotypes 3	2	Barley	Yicheng District, Zhumadian City, Henan Province	114°02′ E; 33°00′ N	2016.04	156	164	214	223	163	163	196	196	94	100	149	158

Biotypes 3	3	Barley	Xincai County, Zhumadian City, Henan Province	115°03′ E; 32°45′ N	2016.04	156	164	217	223	163	163	196	196	94	98	149	158
Biotypes 4	1	Wheat	Balikun County, Hami City, Xinjiang Uygur Autonomous Region	92°53′E; 43°36′N	2016.07	154	164	225	227	152	165	202	202	94	98	139	156
Biotypes 4	2	Wheat	Xiangzhou District, Xiangyang City, Hubei Province	112°14′E; 32°01′N	2016.04	154	166	200	223	159	161	207	207	102	102	208	208
Biotypes 4	3	Wheat	Lianchi District, Baoding City, Hebei Province	115°25′ E; 38°48′ N	2018.04	155	164	216	223	163	163	196	196	94	100	149	158
Biotypes 5	1	Barley	Balikun County, Hami City, Xinjiang Uygur Autonomous Region	92°53′E; 43°36′N	2016.07	164	164	227	227	152	165	202	202	98	98	139	160
Biotypes 5	2	Barley	Balikun County, Hami City, Xinjiang Uygur Autonomous Region	92°53′E; 43°36′N	2016.07	154	166	225	227	152	167	200	204	98	98	139	150
Biotypes 5	3	Wheat	Lianchi District, Baoding City, Hebei Province	115°27′ E; 38°50′ N	2018.04	154	164	223	226	163	165	194	194	94	98	149	158
Biotypes 7	1	Wheat	Lianchi District, Baoding City, Hebei Province	115°27′ E; 38°50′ N	2018.04	156	164	217	223	163	163	196	196	94	100	150	158
Biotypes 7	2	Wheat	Lianchi District, Baoding City, Hebei Province	115°27′ E; 38°50′ N	2018.04	156	164	214	223	163	163	196	196	94	98	149	158
Biotypes 7	3	Wheat	Lianchi District, Baoding City, Hebei Province	115°25′ E; 38°48′ N	2018.04	155	164	223	226	163	163	196	196	94	102	151	158

Table S3. The sequences of primers used in this study.

Gene	Primer	Sequence (5' to 3')
groEL	F	GCCATCCAAAGCCGTATTAGTCA
	R	AGTACCGCAACACCACCAGATA
ef1α	F	TCACCATCATTGACGCACCTG
	R	CCAGTACCAGCAGCAACGATAAG
AtpD	F	CGGGGATCCTGCAGTTTGGWGGWGCWGGWGTWGGWAAAAC
	R	CGGGGATCCGTCGACGCATCWARATGWGCAAAWGTWGTWGCWGG
Gnd	F	CGCGGATCCGGWCCWWSWATWATGCCWGGWGG
	R	CGCGGGCCCGTATGWGCWCCAAAATAATCWCKTTGWGCTTG
16S rDNA	F	AGAGTTTGATCATGGCTCAGATTG
	R	TACCTTGTTACGACTTCACCCCAG
LeuB	F	TAGGTCCTGAAGTTATGCGA
	R	TCCCTGGAAACATCTTGC
IlvD	F	GARTTWGCTGTRAACATWCCWGAACA
	R	GGTAGAGYATCGGTCTCCAA
TrpE	F	CACCCTGACCCTACAATGA
	R	AAGACAACGCCAGCACC

Table S4. Facultative endosymbionts examined in this study.

**Target endosymbiont**	**Target gene**	**Primer**	**Sequence (5' to 3')**
Hamiltonella defensa	*16s rDNA*	F	AGCACAGTTTACTGAGTTCA
		R	TACGGYTACCTTGTTACGACTT
Regiella insecticola	*16s rDNA*	F	ATCGGGGAGTAGCTTGCTAC
		R	CTAGAGATCGTCGCCTAGGTA
Rickettsia sp.	*16s rDNA*	F	AGAGTTTGATCMTGGCTCAG
		R	TCCACGTCACCGTCTTGC
Spiroplasma sp.	*16s rDNA*	F	AGAGTTTGATCMTGGCTCAG
		R	CATCCATCAGCGATAAATCTTTC
Serratia symbiotica	*16s rDNA*	F	AGAGTTTGATCMTGGCTCAG
		R	GCAATGTCTTATTAACACAT
Wolbachia pipientis	*16s rDNA*	F	CATACCTATTCGAAGGGATAG
		R	AGCTTCGAGTGAAACCAATTA

Table S5. Formula of the full-nutrition artificial diet for *Sitobion avenae.*

Ingredient	Mg/100ml	Ingredient	Mg/100ml
L-Met	100.000	Vitamin B7	0.100
L-Gln	600.000	Vitamin B5	5.000
L-Pro	100.000	Choline chloride	50.000
L-Asn	200.000	Folic acid	1.000
L-Glu	200.000	Inositol	50.000
L-Lys	200.000	Nicotinic acid	10.000
L-Arg	400.000	Vitamin B6	2.500
L-Ser	100.000	Vitamin B1	2.500
L-Cyss	1.000	Vitamin C	100.000
L-Tyr	20.000	4-aminobenzoic acid	10.000
L-Trp	150.000	NaCl	1.271
L-Gly	20.000	CuCl2.2H2O	0.300
L-Ile	200.000	FeCl3.6H2O	2.200
L-Ala	29.700	MnCl2.4H2O	0.790
L-His	200.000	ZnCl2	0.471
L-Cys	50.000	Sucrose	30 (g)
L-Phe	100.000	MgCl2.6H2O	150.000
L-Leu	21.400	KH2PO4	200.000
L-Asp	68.400	Cholesterol	5.000
L-Thr	200.000		
DL-Hse	800.000		
L-Val	12.400		

Table S6. Three-way analyses of variance for *Buchnera* abundance affected by host plant, biotype and developmental stage of *Sitobion avenae.*

**Source of variance**	***df***	***F***	***P***	**% total**
Biotype	5	1369.88	< 0.001	23.16
Aphid stage	1	2821.41	< 0.001	47.69
Host plant	5	717.22	< 0.001	12.12
Biotype × Aphid stage	5	147.79	< 0.001	2.50
Biotype × Host plant	25	452.12	< 0.001	7.64
Aphid stage × Host plant	5	234.44	< 0.001	3.96
Biotype × Aphid stage × Host plant	25	171.87	< 0.001	2.91
Error	216	-	-	0.02

Table S7. Comparisons of *Buchnera* abundance in 5-day-old nymphs and adults for six *Sitobion avenae* biotypes.

**Biotypes**	***Buchnera* abundance**		**df**	***t***	***P***
	**5-day old nymphs**	**adults**			
Biotype 1	0.91 ± 0.18	0.46 ± 0.05	46	2.42	0.023
Biotype 2	0.50 ± 0.03	0.39 ± 0.03	46	2.87	0.006
Biotype 3	0.24 ± 0.02	0.14 ± 0.01	46	5.75	< 0.001
Biotype 4	0.30 ± 0.05	0.11 ± 0.02	46	3.60	0.001
Biotype 5	0.60 ± 0.05	0.36 ± 0.03	46	4.07	< 0.001
Biotype 7	0.44 ± 0.04	0.23 ± 0.04	46	3.82	< 0.001

Note: The results are expressed as "mean ± SE".

Table S8. Pearson correlation analyses between 10 d fecundity and *Buchnera* abundance for six biotypes of *Sitobion avenae* (5-day-old nymphs and adults).

**Pearson correlation**	**Biotype 1**	**Biotype 2**	**Biotype 3**	**Biotype 4**	**Biotype 5**	**Biotype 6**
10-day fecundity × *Buchnera* abundance of 5-day-old nymphs	0.889*	0.967**	0.860	0.914*	0.661	-0.041
10-day fecundity × *Buchnera* abundance of adults	0.942*	0.906*	0.583	0.969**	0.418	-0.402

Note: *, $P < 0.05$; **, $P < 0.01$.

Table S9. Principal component analyses (PCA) of 10 d fecundity for six biotypes of *Sitobion avenae* on six varieties before and after rifampin treatments.

Host plant varieties	Before rifampin treatment			After rifampin treatment		
	**PC1**	**PC2**	**PC3**	**PC1**	**PC2**	**PC3**
			**Correlation with principal components**			
Aikang 58	0.241	0.612	0.741	0.623	0.752	0.177
Zhong 4 wumang	0.676	0.623	-0.295	0.944	0.087	-0.062
186-TM12-34	0.319	0.851	-0.118	0.805	-0.418	0.412
Dulihuang	0.884	-0.014	-0.008	0.888	-0.192	-0.262
Zaoshu No.3	0.784	-0.409	0.259	0.910	0.064	-0.138
Xiyin No.2	0.875	-0.271	0.235	0.939	-0.108	-0.028
			**Variance explained in PCA (%)**			
Percentage of principal components (%)	46.30	28.82	12.88	73.76	13.33	4.89
Cumulative percentage (%)	46.30	75.11	88.00	73.76	87.10	91.98

Table S10. Two-way analyses of variance for the developmental times of nymphs and 10 d fecundity for *Sitobion avenae* affected by artificial diets and biotypes.

**Life-history traits**	**Source of variation**	**df**	**Mean square**	***F***	***P***	**% total**
DT1 (d)	Biotypes	5	5.86	26.27	< 0.001	59.95
	Artificial diets	4	2.84	12.73	< 0.001	29.05
	Biotypes × Artificial diets	20	0.851	3.82	< 0.001	8.71
	Error	570	0.22	-	-	2.28
DT2 (d)	Biotypes	5	7.99	35.20	< 0.001	52.88
	Artificial diets	4	6.19	27.27	< 0.001	40.98
	Biotypes × Artificial diets	20	0.70	3.09	< 0.001	4.64
	Error	570	0.23	-	-	1.50
DT3 (d)	Biotypes	5	7.52	25.78	< 0.001	51.39
	Artificial diets	4	5.54	19.01	< 0.001	37.90
	Biotypes × Artificial diets	20	1.28	4.37	< 0.001	8.72
	Error	570	0.29	-	-	1.99
DT3 (d)	Biotypes	5	13.01	36.59	< 0.001	67.13
	Artificial diets	4	4.40	12.36	< 0.001	22.69
	Biotypes × Artificial diets	20	1.62	4.55	< 0.001	8.35
	Error	570	0.36	-	-	1.83
DT (d)	Biotypes	5	67.20	92.46	< 0.001	57.28
	Artificial diets	4	41.91	57.66	< 0.001	35.72
	Biotypes × Artificial diets	20	7.49	10.31	< 0.001	6.39
	Error	570	0.73	-	-	0.62
10-day fecundity	Biotypes	5	152.45	110.50	< 0.001	22.00
	Artificial diets	4	460.36	333.68	< 0.001	66.44
	Biotypes × Artificial diets	20	78.74	57.07	< 0.001	11.36
	Error	570	1.38	-	-	0.20

Note: DT1 to DT4, represent the developmental time of first- to fourth-instar nymphs; DT, represents the total developmental time of nymph.

Table S11. Characters of DNA fragments used in phylogenetic analyses.

Alignment	Aligned sequence length (bp)	Variable sites	Parsimony informative sites	Nucleotide composition T：C：A：G
Gnd	806	10（1.24%）	9（1.11%）	34.9：10.3：39.5：15.3
16S rDNA	1392	——	——	21.1：21.7：28.9：28.3
AtpD	458	19（4.16%）	13（2.84%）	33.4：15.2：30.8：20.7
LeuB	531	11（2.07%）	11（2.07%）	36.8：12.8：33.3：17.2
IlvD	804	7（0.87%）	7（0.87%）	36.3：14.9：34.4：14.4
TrpE	1313	22（1.68%）	21（1.60%）	32.9：13.8：38.6：14.7

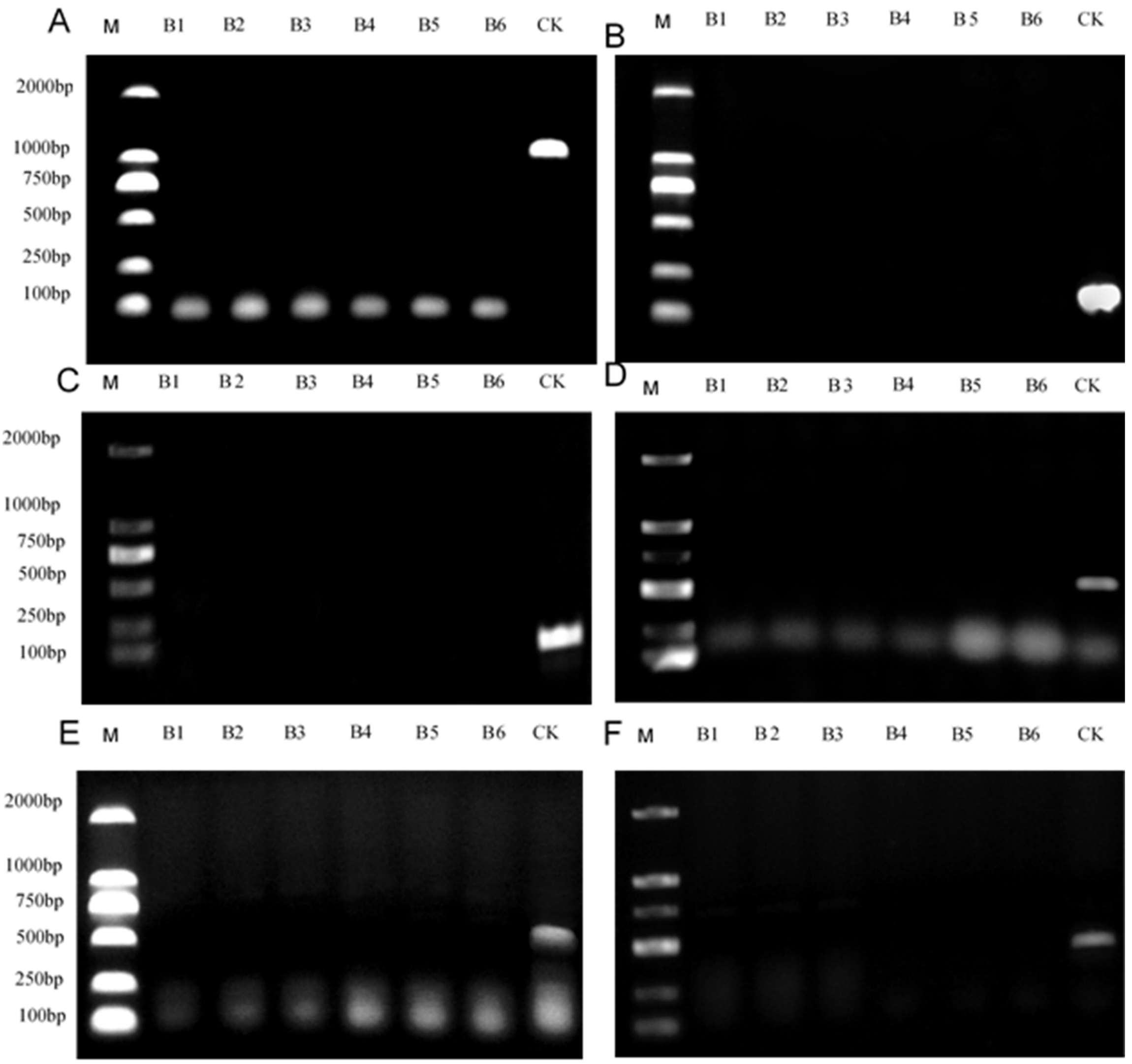

Figure S1. Electrophoretic detection of the facultative endosymbiosis (i.e., A, *Hamiltonella defensa*; B, *Regiella insecticola*; C, *Rickettsia sp.*; D, *Spiroplasma sp.*; E, *Serratia symbiotica*; F, *Wolbachia pipientis*) for clone 3 of six *Sitobion avenae* biotypes. M represents the DNA maker. B1, B2, B3, B4, B5, B6 represent biotype 1, biotype 2, biotype 3, biotype 4, biotype 5, biotype 7, respectively. CK represents the aphid containing the corresponding facultative endosymbiont.

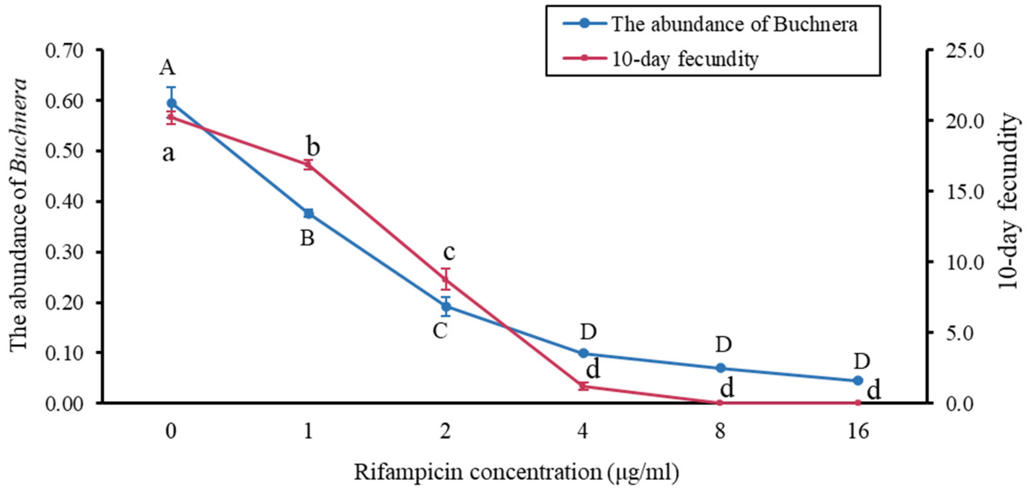

Figure S2. The differences in *Buchnera* abundance and 10 d fecundity of *Sitobion avenae* under rifampicin treatments. Error bars indicate ± SE. Different uppercase and lowercase letters indicate significant differences in *Buchnera* abundance and 10 d fecundity at different rifampicin concentrations (α = 0.05, ANOVA followed by Tukey's tests).

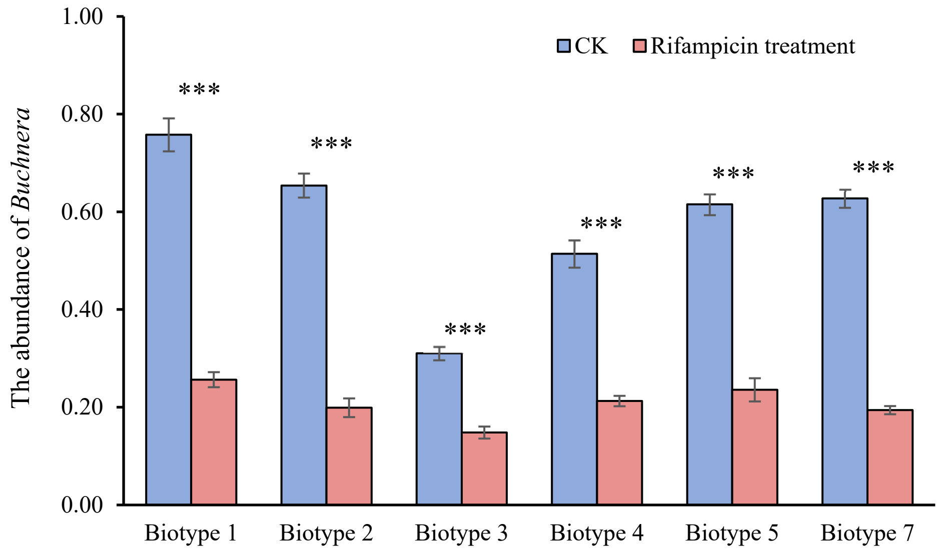

Figure S3. Changes in the abundance of *Buchnera* of six *Sitobion avenae* biotypes under rifampicin treatments.
